# The synergistic effects of traditional Chinese herbs and radiotherapy for cancer treatment

**DOI:** 10.3892/ol.2013.1245

**Published:** 2013-03-12

**Authors:** LILI JIA, SHUMEI MA, XUE HOU, XIN WANG, ABU BAKER LAYTH QASED, XUEFEI SUN, NAN LIANG, HUICHENG LI, HEQING YI, DEJUAN KONG, XIAODONG LIU, FEIYUE FAN

**Affiliations:** 1 Key Laboratory of Radiobiology (Ministry of Health), School of Public Health, Jilin University, Changchun 130021;; 2 Department of Radiation Oncology and Radiology, China- Japan Union Hospital, Changchun 130021; 3 Department of Surgery, Norman Bethune Second Hospital Affiliated to Jilin University, Changchun 130021;; 4 Department of Radiotherapy, Norman Bethune Fourth Hospital Affiliated to Jilin University, Changchun, 130011;; 5 Department of Radiation Hazard Evaluation, Institute of Radiation Medicine of Chinese Academy of Medical Science and Peking Union Medical College, Tianjin 300192, P.R. China

**Keywords:** traditional Chinese medicine, apoptosis, autophagy, radiosensitization, cancer treatment

## Abstract

Traditional Chinese medicine (TCM) has been demonstrated to have potent cytotoxic activity against certain malignant tumors. Ionizing radiation (IR) is one of the most effective methods used in the clinical treatment of cancer. The drawback of a single formula is that it limits the treatment efficacy for cancer, while comprehensive strategies require additional theoretical support. However, a combination of different antitumor treatment modalities is advantageous in restricting the non-specific toxicity often observed with an extremely high dose of a single regimen. The induction of apoptotic cell death is a significant process in tumor cells following radiotherapy or chemotherapy, and resistance to these treatments has been linked to a low propensity for apoptosis. Autophagy is a response of cancer cells to IR or chemotherapy, and involves the prominent formation of autophagic vacuoles in the cytoplasm. In this review, the synergistic effects of TCM and radiotherapy are summarized and the underlying mechanisms are illustrated, providing new therapeutic strategies for cancer.

## Contents

1. Introduction
2. Application of TCM in cancer
3. TCM and radiotherapy
4. TCM, radiosensitivity and apoptosis
5. TCM, radiosensitivity and autophagy
6. Prospects

## 1. Introduction

Ionizing radiation (IR) is widely used in cancer therapy and can induce cell cycle arrest, DNA repair and repair, and/or apoptosis, leading to different biological consequences depending on the cell type. Traditional Chinese medicine (TCM) is often considered to be an alternative or complementary medicine in cancer treatment; it is able to suppress tumor growth and angiogenesis, as well as inhibit invasion and metastasis (1–10). TCMs may also function as a radiosensitizers during the radiotherapy of cancer.

Apoptosis, or type I programmed cell death, is a major response of cancer cells to various therapies including IR and TCM (28–56). Apoptosis is controlled by extrinsic or intrinsic inducers, which often act as targets of TCM to trigger cell death during radiotherapy. Autophagy (type II programmed cell death), a protein degradation system involving the autophagic/lysosomal compartment, is another response of cancer cells to various therapies. The process of autophagy is initiated by the sequestration of a portion of the cytoplasm to form the autophagosome. Subsequently, the autophagosome fuses with the lysosome and the contents of the autophagosome are lyzed. Activation of autophagy in the tumor cells may improve the outcome of radiotherapy. The roles of TCM in radiation-induced cell death determine the application of TCM in cancer treatment.

## 2. Application of TCM in cancer

TCM has been used for thousands of years. The majority of Chinese herbal formulae comprise several herbal components and have been used to treat various chronic diseases, mainly by immunomodulation or by altering inducible cell death. Although TCM has assigned anticancer activities, the underlying mechanisms are not well understood. Certain herbs are able to inhibit the migration and invasion of cancer cells *in vitro*, while others trigger apoptosis in cancer cells. The herbs used in TCMs for treating human cancer are summarized in Table I (1–10).

## 3. TCM and radiotherapy

A series of studies concerning the radioprotective and radiosensitizing functions of TCM have been conducted over a long time period. In the 20th century, scientists focused on testing the radioprotective effects of TCM in experimental animals. A series of bioactive components were isolated from TCMs and the mechanisms of radioprotection were studied. Improving the function of the hematopoietic and immune systems may be characteristic of such TCM herbs.

Kuei-Pi-Tang is a type of TCM that has been suggested to have therapeutic effects on hemato-deficient diseases and radiation-related injuries. Kuei-Pi-Tang has been demonstrated to increase the recovery of cellular immunocompetence, particularly when administered at a concentration of 20 mg/20 g body weight following γ-ray irradiation (11). Additionally, Ren-Shen-Yang-Rong-Tang, Zaizhang-I (ZZ-I) and Juzen-Taiho-Toh (TJ-48) have demonstrated effects on the hematopoietic recovery from radiation-related injury in mice, by stimulating hematopoietic stem cells and by improving the hematopoietic inductive microenvironment (HIM). The results demonstrated that these TCM herbs significantly promoted the recovery of the colony-forming unit-spleen (CFU-S) and the colony-forming unit-granulocyte/macrophage (CFU-GM) (12–14). Furthermore, *Gynostemma pentaphyllum* (Gp) has been shown to assist in the recovery of decreased leukocyte counts, GOT, GPT and IgG serum levels, and the proliferation of splenocytes stimulated by PHA, LPS and Con A in γ-ray-irradiated mice (15). Moreover, Dang-Gui-Shao-Yao-San (DGSYS), administered to previously X-irradiated mice increased the number of CFU-S in the mice that survived the irradiation, as well as significantly ameliorating leukopenia, thrombocytopenia and the depression of hematocrits following irradiation (16). In addition, certain TCMs have been demonstrated to increase immunocompetence in γ-ray-irradiated mice; *Ganoderma lucidum* (GI) and Krestin (PSK) increased the splenic weight and leukocyte count following γ-ray irradiation (17). Furthermore, Si-Wu-Tang was observed to protect the jejunal crypts, increase the formation of endogenous spleen colonies and reduce the frequency of radiation-induced apoptosis when administered prior to irradiation; while extracts of Danggui and Baishaoyao have been revealed to have potentially significant radioprotective effects (18).

Throughout the 21st century, numerous scientists have begun to administer TCM herbs as an adjunct to radiotherapy/chemotherapy for certain types of cancer. We discuss examples of such herbs used in radiotherapy. TCM has been shown to exhibit radiomodifying effects on tumors and normal tissues by *in vitro* and *in vivo* studies. A number of these agents are able to enhance the therapeutic gain of radiotherapy by acting as radiosensitizers to the tumor cells and/or as radioprotectors to normal cells. Botanical agents are comprised of multiple phytochemical compounds that may work individually or synergistically to not only improve the outcomes of radiotherapy, but also to induce a variety of anticancer effects. It is important to evaluate these botanical agents for their efficacy, tumor specificity and safety profiles during radiotherapy (19). The goji berry, *Lycium barbarum*, is well-recognized in TCM for its various therapeutic properties based on its antioxidant and immunomodulating effects. A previous study demonstrated the antioxidant activity of the goji berry in the skin; 5% goji berry juice provided significant protection against lipid peroxidation induced by UVA radiation. Two known inducible endogenous skin antioxidants, haem oxygenase-1 and metallothionein, were found to be involved in the photoimmune protection. Therefore, goji berry juice has the potential to provide additional photoprotection for susceptible humans (20). Studies have also demonstrated that TCM herbs are able to protect hematopoietic organs against radiation-induced damage. For example, panaxatriol (PT) has been observed to relieve myelosuppression induced by radiation injury. The ability of the agent to regulate the expression of the hematopoietic growth factor GM-CSF and to promote the maturation of bone marrow cells may be responsible for a number of these beneficial effects (21). Additionally, the effects of the main ingredients of Bu-Zhong-Yi-Qi-Tang on jejunal crypt survival, endogenous spleen colony formation and apoptosis in jejunal crypt cells were investigated in mice irradiated with high and low doses of γ-rays (22).

IR is capable of inducing DNA damage and cell death by generating reactive oxygen species (ROS). Pre-treatment of thymocytes with paeoniflorin (PF) has been demonstrated to reverse this tendency and to attenuate irradiation-induced ROS generation. Several antiapoptotic characteristics of PF, including the ability to diminish cytosolic Ca^2+^ concentration, inhibit caspase-3 activation, upregulate Bcl-2 and downregulate Bax in 4-Gy-irradiated thymocytes, have been identified. PF was also observed to block extracellular signal-regulated kinase (ERK), c-Jun N-terminal kinase (JNK) and p38 kinase, which were activated by 4-Gy irradiation (23). Furthermore, in human dermal fibroblasts, berberine (BBR) was able to decrease UV-induced MMP-1 expression and reverse UV-induced reduction of type I procollagen in a dose-dependent manner (24). Additionally, an isoflavone extract from soybean cake decreased UVB-induced HaCaT cell death and the phosphorylation of p38, JNK and ERK1/2 *in vitro*. In the *in vivo* studies, topical application of isoflavone extract prior to UVB irritation decreased the epidermal thickness and the expression levels of COX-2 and PCNA, and increased the catalase concentration (25). Moreover, the Chinese herbal preparation, Yangyin Humo Decoction (YHD), was able to alleviate the oral mucomembranous reaction to radiation applied to patients with head-neck tumors (26). In addition, aloe polysaccharides (AP) have demonstrated radioprotective effects by blocking the upregulation of pro-apoptotic p53, Bax and Bad, and the downregulation of Bcl-2, in normal human cells *in vitro* and in mice *in vivo*(27).

## 4. TCM, radiosensitivity and apoptosis

Apoptosis is one of the processes of programmed cell death (PCD) that occurs in multicellular organisms, where biochemical events lead to characteristic cell changes and death. In addition to the significance of apoptosis as a biological phenomenon, defective apoptotic processes have been implicated in numerous types of diseases. Excessive apoptosis causes atrophy, as in the case of ischemic damage; whereas an insufficient level of apoptosis results in uncontrolled cell proliferation, such as in cancer. TCMs act in cancer via various effects and pathways (Tables II, III and IV) (28–56).

Radiosensitive cells have been demonstrated to correlate with a good outcome following radiotherapy. An increased understanding of the molecular processes underlying cellular sensitivity to IR has led to the identification of novel targets for intervention. A combination of a monoclonal antibody (TRA-8) to the human death receptor (DR5) and IR enhanced radiosensitivity in the human radioresistant larynx squamous carcinoma cell line, providing an effective treatment strategy for patients with radioresistant cancers (57). Additionally, wortmannin combined with X-rays inhibited DNA-dependent protein kinase (DNA-PK), resulting in the inhibition of double-strand break (dsb) repair in the fast component. This effect enhanced the induction of apoptosis during the radiosensitization of bladder tumors (58). Moreover, increased levels of heat shock protein (Hsp) 27 and 70 have been identified to be closely correlated with tumorigenesis, metastasis, resistance to anticancer therapeutics and thus a poor prognosis in a wide range of tumors. Silencing of Hsp27 and Hsp70 has both enhanced radiation sensitivity and amplified irradiation-induced apoptosis (59). Certain agents have demonstrated their ability to inhibit antiapoptotic proteins of the Bcl-2 and caspase families on exposure to radiation, thus enhancing the radiation sensitivity. Exploration of the clinical applications of these agents as radiosensitizers for tumor therapy is merited (60–68). Simultaneous inhibition of STAT3 and ErbB2 has been demonstrated to induce U251 cell apoptosis, which was primarily associated with the mitochondrial apoptotic pathway and radiosensitizing activity in human glioma (69).

The HDAC inhibitor, valproic acid (VPA), which enhances IR-induced mitochondrial localizations of Bax and Bcl-xL, was observed to upregulate the mitochondrial membrane potential and be involved in the release of cytochrome *c* only in wild-type p53 cell lines. It was also found to enhance the radiotherapy response in colorectal cancer, particularly in tumors with the wild-type p53 genotype (70). p53-upregulated modulator of apoptosis (PUMA), is a Bcl-2 homology 3 (BH3)-only Bcl-2 family member that directly binds and antagonizes all known antiapoptotic Bcl-2 family members to induce mitochondrial dysfunction and caspase activation (71). Additionally, Ser46 phosphorylation of p53 has been demonstrated to induce coincident caspase-7 and PARP cleavage in response to IR. Furthermore, mutation of p53 (Ser46) to alanine attenuated IR-induced apoptotic signaling, and ought, therefore, to be a target for radiosensitization (72). Moreover, inhibition of Bcl-xL expression has been observed to result in potent antitumor activity and radiosensitization in human prostatic carcinoma (73).

Certain TCM herbs act as radioprotective or radiosensitizing agents via apoptotic pathways. Treatment with a combination of arsenic trioxide and irradiation has been shown to enhance the apoptotic effects in U937 cells through increased mitotic arrest and ROS generation accompanied by a decrease in Bcl-2 and Bcl-xL levels, and upregulation of caspase-3 levels (74). In addition, a water-soluble ginseng (*Panax* root) extract was observed to provide greater protection against radiation-induced DNA damage than isolated ginsenoside fractions (saponins). The underlying radioprotective mechanism of ginseng may be linked, directly or indirectly, to its antioxidative capability by the free radicals responsible for DNA damage. Ginseng is considered to be a promising radioprotector in therapeutic or preventive protocols that is able to attenuate the deleterious effects of radiation in human normal tissue, particularly in cancer patients undergoing radiotherapy (75).

## 5. TCM, radiosensitivity and autophagy

Autophagy, also known as type II programmed cell death or autophagocytosis, is a catabolic process involving the degradation of cellular components through the lysosomal machinery. It is a tightly regulated process that occurs naturally in cell growth, development and homeostasis, and assists in maintaining a balance between the synthesis, degradation and subsequent recycling of cellular products. It is a key mechanism by which a starving cell reallocates nutrients from unnecessary processes to more essential processes. It is unknown whether the autophagic activity in dying cells itself causes death or whether it simply occurs as a process alongside it. A cell may either die or survive, and these two outcomes are dependent on environmental factors. It is unclear whether the increase in autophagosomes indicates an increase in autophagic activity or a decrease in autophagosome-lysosome fusion (76).

Many signal pathways participate in the process of autophagy. Mammalian target of rapamycin (mTOR) senses nutrient, metabolic and hormonal signals and is involved in numerous regulatory events associated with energy metabolism, including the nuclear localization of nutrient-regulated transcription factors. mTOR is abundantly expressed when nutrients are plentiful and it suppresses autophagy. Phosphoinositide-3-kinase I (PI3KI)/AKT is an upstream regulator of mTOR and p70S6 kinase is the downstream effector of mTOR, PI3KI/AKT-mTOR-p70S6 kinase signaling represses the process of autophagy. Additionally, Beclin 1 represents an important component of the autophagic machinery; it interacts with proteins that positively regulate autophagy, such as Vps34, UVRAG and Ambra1, as well as with antiapoptotic proteins such as Bcl-2 (via its BH3-like domain) to negatively regulate autophagy (77). Beclin 1 acts as a part of the class III PI3K (PI3KIII) Vps34 complex that induces autophagy (78). The Bcl-2 families are also regulators of autophagy; Bcl-xL, which normally protects cells from autophagy by inhibiting the Beclin-1/Vps34 complex, is essential for autophagy (79). Bax and Bak, which act as a gateway for caspase-mediated cell death, also function as downregulators of autophagy (80).

Several regulators of the apoptotic pathway, such as PI3K/Akt signaling, p53 and Bcl-2 family members, also function as modulators of autophagy. Taking Bcl-2 as an example, Beclin 1 is capable of binding to PI3KIII as well as to Bcl-2, and the Bcl 2-Beclin complex inhibits the process of autophagy, while the dissociation of Beclin 1 from its Bcl-2 inhibitor is essential for its autophagic activity. DAPK is able to phosphorylate Beclin 1 at Thr119, which is located at a crucial position within its BH3 domain, and thus promote the dissociation of Beclin 1 from Bcl-xL and the induction of autophagy (81). Therfore, there may be a ‘molecular switch’ between apoptosis and autophagy that has these pathways in common.

The theory that TCM plays an active role in cancer treatment by inducing autophagy has been studied. The medicinal mushroom, *Ganoderma lucidum,* is one of the most esteemed natural products that has been used as a TCM. *G. lucidum* triterpene extract (GLT) has been demonstrated to suppress the phosphorylation of p38 MAPK, and to induce autophagy and Beclin 1 expression in colon cancer cells (82). In addition, pheophorbide-a (Pa) is an active component isolated from a Chinese herb and Pa-based photodynamic therapy (Pa-PDT) has demonstrated antitumor effects by activating mitochondria-mediated apoptosis and ERK-mediated autophagy in MDA-MB-231 cells (83). Moreover, as mTOR plays a central role in the autophagy pathway, TCMs that are able to induce tumor cell death through mTOR may potentially induce autophagy (Table V). Other TCMs mentioned in Table IV may also potentially participate in the regulation of autophagy. Furthermore, flavokawain B, a novel chalcone from *Alpinia pricei* Hayata with potent anticancer activity, significantly inhibits the growth of colon cancer cells, thus providing novel insights into the molecular mechanisms underlying its apopototic activity. Flavokawain B provokes G2/M accumulation in addition to autophagy, and it also acts through ROS generation and GADD153 upregulation to regulate the expression of Bcl-2 family members, thereby inducing mitochondrial dysfunction and apoptosis in HCT116 cells (84). Furthermore, licorice, a common Chinese medicinal herb with antitumor activity, is able to induce autophagy by downregulating Bcl-2 and inhibiting the mTOR pathway (85).

Previously, it was considered that cell death induced by IR is apoptotic, while autophagy is an alternative cell death pathway that is induced by mTOR inhibitors and upregulated when apoptosis is defective. The novel response of cancer cells to IR or chemotherapy has been demonstrated to increase the resistance of cancer cells to various apoptotic stimuli. Upregulation of autophagy by inhibitors of caspase-3 and mTOR enhanced radiotherapy responses in a mouse model of lung cancer. Combined Bcl-2/mTOR inhibition was observed to lead to enhanced radiosensitivity via induction of apoptosis and autophagy *in vitro* and in a lung cancer xenograft model; this is a potential therapeutic strategy for enhancing radiation therapy in patients with non-small cell lung cancer. Bromodeoxyuridine (BrdU) was found to enhance and modify radiation-induced cell death by accelerating the increase in the Bax/Bcl-2 ratio in non-irradiated cells, subsequently increasing radiation-induced apoptosis and/or autophagy depending on the radiation dosage (76,79–81). Moreover, the cell wall skeleton of *Mycobacterium bovis* bacillus Calmette-Guérin (BCG/CWS) is an effective antitumor immunotherapy agent; BCG/CWS plus IR-induced autophagy and cell death were predominantly mediated by the generation of ROS. This suggests that BCG/CWS in combination with IR is a promising therapeutic strategy for enhancing radiation therapy in colon cancer cells through the induction of autophagy (82). However, once a tumor has formed, autophagy inhibition may be a therapeutic target for radiosensitization and chemosensitization. At present, the relationship between cancer and deregulated autophagy appears to be complex and must be disentangled by further in-depth study (83).

Z-VAD, a broad-spectrum caspase inhibitor, is a radiosensitizer in breast and lung cancer *in vitro* and *in vivo*. Caspase inhibition is proposed to have the potential to enhance the therapeutic ratio of radiation therapy in solid tumors. Therefore, clinical trials are required to determine the potential of this combination therapy in cancer patients. In addition, the autophagy inhibitors, 3-methyladenine (3-MA) and bafilomycin A1, may represent a new application of radiosensitization for malignant glioma cells (85,86). Moreover, vitamin D or vitamin D analogs are involved in the radiosensitization of breast tumor cells mediated via autophagy, and also delay and attenuate the proliferative recovery that may be a preclinical indicator of disease recurrence (84). Furthermore, DNA-PK plays a key role in the DNA DSB repair induced by IR; inhibition of DNA-PK combined with IR is capable of inducing autophagy and radiosensitizing the malignant glioma cells, and may be promising as a new therapy for radiosensitizing tumors. Additionally, pharmacological inhibition of nuclear factor (NF)-κB has been demonstrated to enhance cell damage and radiosensitization through autophagy in glioma cell lines (87,88).

## 6. Prospects

Currently, the functions of TCMs in cancer cells and the relative underlying molecular mechanisms are not yet completely understood. The studies described in this review have indicated that TCMs, which are observed to have a radiosensitizing effect through mechanisms involving their apoptotic and autophagic properties, have the potential to be effective systemic radiosensitizers that may be used to amplify radiation-induced toxicity in tumor tissues. TCMs are able to exhibit anticancer roles by apoptosis and autophagy, through mTOR and the Bcl-2 family pathway. Although the molecular mechanisms underlying autophagy are not yet fully understood, modulators of autophagy may have a number of potential benefits; promoters of autophagy may induce autophagy-mediated cell death in types of cancer with a high threshold to apoptosis. Certain TCM herbs may be used as radioprotectors that are able to ameliorate radiation-induced toxicity in normal tissue in cancer patients undergoing radiotherapy. We propose further evaluation of TCM for its radiosensitive and radioprotection potential in a clinical setting.

## Figures and Tables

**Table I:** Summary of reported effects on human cancer following TCM administration.

TCM	Cancer	Effect	Pathway	Reference
Yanhusuo	Breast cancer	Inhibits migration and invasion	Activates p38; inhibits ERK1/2 and ASPK/JNK (MAPKs)	(1)
Celastrol	Prostate cancer	Suppresses tumor growth and angiogenesis	AKT/mTOR/p70S6K	(2)
Icariin	Gastric cancer	Inhibits migration and invasion	Rac1-dependent VASP pathway	(3)
Tanshinone IIA	Cervical cancer	Inhibits invasion and metastasis	G2/M arrest and subsequent apoptosis	(4,5)
	Hepatocellular cancer	Inhibits invasion and metastasis	Inhibits MMP2,MMP9 and NF-κB	
Berbamine	Lung cancer	Suppresses growth and migration	Reduces Bcl-2/Bax protein ratio	(6)
GSPP	Hepatoma	Inhibits proliferation and migration	S phase arrest; calcium-mediated regulation of the actin cytoskeleton reorganization	(7)
Gecko	Esophageal cancer	Inhibits growth	Apoptosis; downregulation of VEGF and bFGF protein expression	(8)
Ganodema	Colorectal cancer	Inhibits growth	G2/M arrest	(9)
DATS	Osteosarcoma cancer	Suppresses cell proliferation	G0/G1 phase arrest; induces apoptosis	(10)

TCM, traditional Chinese medicine; GSPP, gekko sulfated polysaccharide-protein complex; DATS, diallyl trisulfide; ERK, extracellular signal-related kinase; ASPK, aspartate kinase; JNK, c-Jun N-terminal kinase; MAPK, mitogen-activated protein kinase; AKT, v-akt murine thymoma viral oncogene homolog 1; mTOR, mammalian target of rapamycin; P70S6K, ribosomal protein S6 kinase, 70kDa, polypeptide 1; Rac-1, Ras-related C3 botilinum toxin substrate 1; VASP, vasodilator-stimulated phosphoprotein; MMP2, matrix metallopeptidase 2; MMP9, matrix metallopeptidase 9; NF-κB, nuclear factor of κ light polypeptide gene enhancer in B-cells; Bcl-2, B-cell CLL/lymphoma 2; Bax, Bcl-2-associated X protein; VEGF, vascular endothelial growth factor; bFGF, basic fibroblast growth factor.

**Table II:** Summary of reported actions of TCM in apoptosis through Fas/FasL.

TCM	Cancer	Effect	Pathway	Reference
KLT	Hepatoma	Apoptosis	Fas/FasL; G2/M arrest	(28)
Berbamine	Hepatoma	Apoptosis	Fas/FasL	(29)
Waltonitone	Hepatoma	Inhibits growth; apoptosis	Fas/FasL; mitochondria pathway	(30)
Celastrol	Breast cancer	Apoptosis	TRAIL	(31)
Cantharidin	Pancreatic cancer	G2/M arrest; apoptosis	JNK	(32)
Genistein	Hepatoma	Apoptosis	Fas/FasL; mitochondria pathway	(33)

TCM, traditional Chinese medicine; KLT, Kang-Lai-Te; Fas, TNF receptor superfamily; FasL, Fas ligand; TRAIL, TNF-related apoptosis-inducing ligand; JNK, c-Jun N-terminal kinase.

**Table III:** Summary of reported actions of TCM in apoptosis through the mTOR pathway.

TCM	Cancer	Effect	Pathway	Reference
Osthole	Breast cancer	Inhibits proliferation	AKT/mTOR; induces apoptosis	(34)
SYUNZ-16	Hepatoma	Inhibits growth Apoptosis	PKB/AKT AKT/FOXO	(35)
Casticin	Leukemia	Apoptosis	PI3K/AKT	(36)
AECM	Breast cancer	Apoptosis	AKT	(37)
Antroquinonol	Hepatoma	G1 arrest; apoptosis	AMPK mTOR	(38)

TCM, traditional Chinese medicine; AECM, aqueous extract of *Cordyceps militaris*; AKT, v-akt murine thymoma viral oncogene homolog 1; mTOR, mammalian target of rapamycin; PKB, protein kinase B; FOXO, forkhead box; PI3K, phosphoinositide-3-kinase.

**Table IV:** Summary of reported actions of TCM in apoptosis through the Bcl-2 family.

TCM	Cancer	Effect	Pathway	Reference
*Toona sinensis*	Lung cancer	G1 arrest; apoptosis	Bcl-2	(39)
ApoG2	Hepatoma	Apoptosis	Bcl-2; Bcl-xL; caspase-3	(40)
Baicalin	Breast cancer	G0/G1 arrest; apoptosis	Bax	(41)
As_2_O_3_	Neuroblastoma	Apoptosis	Bcl-2; Bid; Bcl-xL	(42)
Bufotalin	Hepatoma	G1 arrest; apoptosis	AIF caspase	(43)
GA3	Leukemia	Apoptosis	Bcl-2; Bax	(44)
GA	Malignant melanoma	Apoptosis	Bcl-2; Bax; caspase-3	(45)
ON-III	Breast cancer	Apoptosis	Bim	(46)
Rhein	Tongue cancer	S arrest; apoptosis	Bcl-2; Bax; caspase-3	(47)
PSI	Ovarian cancer	G2/M arrest; apoptosis	Bcl-2; Bax; caspase-3 and -9	(48)
Corosolic	Cervix adenocarcinoma	S arrest; apoptosis	Bcl-2; Bax; caspase-3,-8 and -9	(49)
*Alpinia pricei* rhizome	Oral epithelium carcinoma	Apoptosis	Bcl-2; Bax; caspase-3 and -9	(50)
Cinobufacini	Hepatoma	Apoptosis	Bcl-2; Bax; caspase-3 and -9; mitochondria pathway	(51)
*Houttuynia cordata* Thunb	Colon adenocarcinoma	Apoptosis	Bcl-2; Bax; mitochondria pathway	(52)
DHA	Lung cancer	Apoptosis	Caspase-3	(53)
Plumbagin	Pancreatic cancer	Apoptosis	Bcl-2; Bax; caspase-3,-8 and -9	(54)
CPBF	Cervix adenocarcinoma	Apoptosis	Bcl-2; Bax; caspase-3 and -9; mitochondria pathway	(55)
ART	T leukemia	Apoptosis	Bcl-2; ROS	(56)

TCM, traditional Chinese medicine; ApoG2, apogossypolone; As_2_O_3_, arsenic trioxide; GA3, gambogic acid 3; GA, gambogic acid; ON-III, 2′,4′-dihydroxy-6′-methoxy-3′,5′-dimethylchalcone; PSI, Paris Saponin I; DHA, dihydroartemisinin; CPBF, *Cordyceps pruinosa* butanol fraction; ART, artesunate; Bcl-2, B-cell CLL/lymphoma 2; Bcl-xL, apoptosis regulator Bcl-X; Bax, Bcl-2-associated X protein; Bid, BH3-interacting domain death agonist; AIF, apoptosis inducing factor; Bim, Bcl-2-like 11; ROS, c-ros oncogene.

**Table V:** Summary of reported actions of TCM in tumors through the mTOR pathway, by inducing or potentially inducing autophagy.

TCM	Cancer	Effect	Pathway	Reference
Licorice	Prostate	Induces autophagy	mTOR	(85)
Alisol B	Gastrc	Induces autophagy	CaMKK-AMPK-mTOR	(86)
Osthole	Breast	Inhibits proliferation; induces autophagy	AKT/mTOR	(34)
SYUNZ-16	Hepatoma	Inhibits growth autophagy	PKB/AKT; AKT/FOXO	(35)
Casticin	Leukemia	Autophagy	PI3K/AKT	(36)
AECM	Breast	Autophagy	AKT	(37)
Antroquinonol	Hepatoma	G1 arrest; autophagy	AMPK; mTOR	(38)

TCM, traditional Chinese medicine; AECM, aquenous extract of *Cordyceps militaris*; mTOR, mammalian target of rapamycin; CaMKK, calcium/calmodulin-dependent protein kinase kinase; AMPK, AMP-activated protein kinase; AKT, v-akt murine thymoma viral oncogene homolog 1; PKB, protein kinase B; FOXO, forkhead box; PI3K, phosphoinositide-3-kinase.
